# The Association Between Arterial Stiffness and Muscle Indices Among Healthy Subjects and Subjects With Cardiovascular Risk Factors: An Evidence-Based Review

**DOI:** 10.3389/fphys.2021.742338

**Published:** 2021-11-23

**Authors:** Amilia Aminuddin, Muhammad Fakhrurrazi Noor Hashim, Nur Aina Syazana Mohd Zaberi, Lee Zheng Wei, Beh Ching Chu, Nur Amalina Jamaludin, Norizam Salamt, Nur Aishah Che Roos, Azizah Ugusman

**Affiliations:** ^1^Department of Physiology, Faculty of Medicine, Universiti Kebangsaan Malaysia Medical Centre, Cheras, Malaysia; ^2^Faculty of Medicine and Defence Health, National Defence University of Malaysia, Kem Sungai Besi, Malaysia

**Keywords:** arterial stiffness, pulse wave velocity, muscle mass, muscle strength, muscle flexibility, cardiovascular

## Abstract

Skeletal muscle is one of the major tissues in the body and is important for performing daily physical activity. Previous studies suggest that vascular dysfunction contributes to reduced skeletal muscle mass. However, the association between vascular dysfunction and muscle mass, muscle strength and muscle flexibility are less established. Therefore, the focus of this review was to investigate the association between arterial stiffness (AS) which is a marker of vascular function, and muscle indices among healthy and those with cardiovascular risk factors. Three databases were used to search for relevant studies. These keywords were used: “arterial stiffness” OR “vascular stiffness” OR “aortic stiffness” OR “pulse wave velocity” OR “carotid femoral pulse wave velocity” OR “pulse wave analysis” AND “muscle” OR “skeletal” OR “flexibility” OR “range of motion” OR “articular” OR “arthrometry” OR “strength” OR “hand strength” OR “pinch strength” OR “mass” OR “lean” OR “body composition.” The criteria were; (1) original, full-text articles, (2) articles written in English language, (3) human studies involving healthy adults and/or adults with cardiovascular disease (CVD) or CVD risk factors (4) articles that reported the relationship between AS (measured as carotid-femoral pulse wave velocity or brachial-ankle pulse wave velocity) and muscle indices (measured as muscle mass, muscle flexibility and muscle strength) after adjusting for relevant confounders. The search identified 2295 articles published between 1971 and June 2021. Only 17 articles fulfilled the criteria. Two studies showed an inverse association between AS and muscle strength in healthy subjects, whereas in subjects with CVD risk factors, five out of seven studies found an inverse correlation between the two parameters. Eleven studies showed an inverse association between AS and muscle mass in subjects with CVD and CVD risk factors. The association between AS and muscle flexibility was not studied in any of the articles reviewed. In conclusion, there is an inverse correlation between muscle indices and AS in healthy adults and those with CVD or CVD risk factors. However, most of the studies were cross-sectional studies, hence the need for future prospective studies to address this issue.

## Introduction

Skeletal muscle comprises about 40% of the body weight and is important for performing daily physical activity (Sherwood, 2008). A good muscle strength and flexibility may help to reduce the risk of injury and fall which could lead to physical disabilities, poor quality of life and mortality. Muscle function is also related with muscle mass (Reed et al., 1991). Muscle receives about 15% of cardiac output at rest, and the need for blood supply increases during exercise (Sherwood, 2008). Thus, a good blood supply is important for muscle to function efficiently. The role of vascular function in the development of muscle mass was addressed in a recent study by Jeon et al. (2021). It was observed that poor vascular function may impair oxygen and nutrient delivery to the muscles, hence causing impairment of muscle protein synthesis and alteration in mitochondrial function (Groen et al., 2014; Jeon et al., 2021).

In human research, one of the non-invasive methods to assess vascular function is by measuring arterial stiffness (AS). AS is described as an elastic resistance to deformation that involves complex interactions between the extracellular matrix components such as elastin, collagen, glycoproteins and proteoglycans, and vascular smooth muscle cells in the arterial wall (Safar et al., 2003). AS is accelerated by aging and classic CVD risk factor such as atherosclerosis, thus reducing the normal arterial compliance. Stiffness of the aorta is frequently studied and the gold standard measurement of aortic stiffness is carotid-femoral pulse wave velocity (cfPWV), which measures the speed of the pressure wave that travels from the aorta to the femoral artery (Laurent et al., 2006). Another marker of AS is brachial-ankle PWV (baPWV) which indicates central and peripheral arterial stiffness (Katakami et al., 2014). PWV was independently associated with cardiovascular events as highlighted by the European Society of Cardiology/European Society of Hypertension Guidelines (Mancia et al., 2013).

Previous studies have investigated the association between PWV and muscle indices (Watson et al., 2011; van Dijk et al., 2015; Lima-Junior et al., 2019). A prospective study showed that increased PWV was associated with poor muscular function in people with altered blood flow (Watson et al., 2011). It was also revealed that reduction in muscle flexibility and strength was an indicator of increased arterial stiffness (AS) (Yamamoto et al., 2009; Fahs et al., 2010). However, several studies showed no significant association (van Dijk et al., 2015; Lima-Junior et al., 2019). This might be contributed by different methods of measurements and low sample sizes. Hence, the objective of this study was to investigate the relationship between AS and muscle indices by systematically reviewing the relevant studies involving healthy adults and those with established CVD or CVD risk factors, which were known to affect vascular function.

## Materials and Methods

### Search Strategy

The literature search was conducted up to June 2021 based on three databases: Web of Science, PubMed and Scopus. The following keywords were used as search strategy: (“aortic stiffness”) OR (“arterial stiffness”) OR (“vascular stiffness”) OR (“pulse wave velocity”) OR (“carotid femoral pulse wave velocity”) OR (“pulse wave analysis”) AND (“skeletal”) OR (“muscle”) OR (“range of motion”) OR (“flexibility”) OR (“arthrometry”) OR (“articular”) OR (“strength”) OR (“pinch strength”) OR (“hand strength”) OR (“body composition”) OR (“lean”) OR (“mass”).

### Selection Criteria

Articles that that have been extracted using the keywords were screened by two authors (BC and NJ). The criteria used were (1) original, full-text articles, (2) articles written in English language, (3) human studies involving healthy adults and/or adults with established CVD or CVD risk factors (4) articles that reported the relationship between AS (measured as cfPWV or baPWV) and muscle indices (measured as muscle mass, muscle flexibility and muscle strength) after adjusting for relevant covariates or confounders. Adjustment for covariates is necessary since there are several factors that influence PWV such as age, blood pressure, heart rate and CVD risk factors such as hypertension, dyslipidemia and diabetes mellitus. Studies involving subjects with chronic lung, kidney, inflammatory diseases and malignancies were excluded. We also excluded studies that used simple correlation for the associations.

### Article Screening

In this study, the articles were screened in three phases. Initially, the articles were omitted in view of their title and keywords. Next, after reviewing the abstracts, the articles that did not follow the criteria were omitted. In the final phase, articles that were not related to the association between AS and muscle indices were omitted after reading the full text. The details of the studies were summarized in a table which included the design of the study, subjects’ characteristic, mean age, male percentage, method of measurement and the findings. The selected studies were divided into two categories; (1) studies involving healthy subjects or (2) studies involving subjects with established CVD or CVD risk factors.

## Results

**FIGURE 1:**
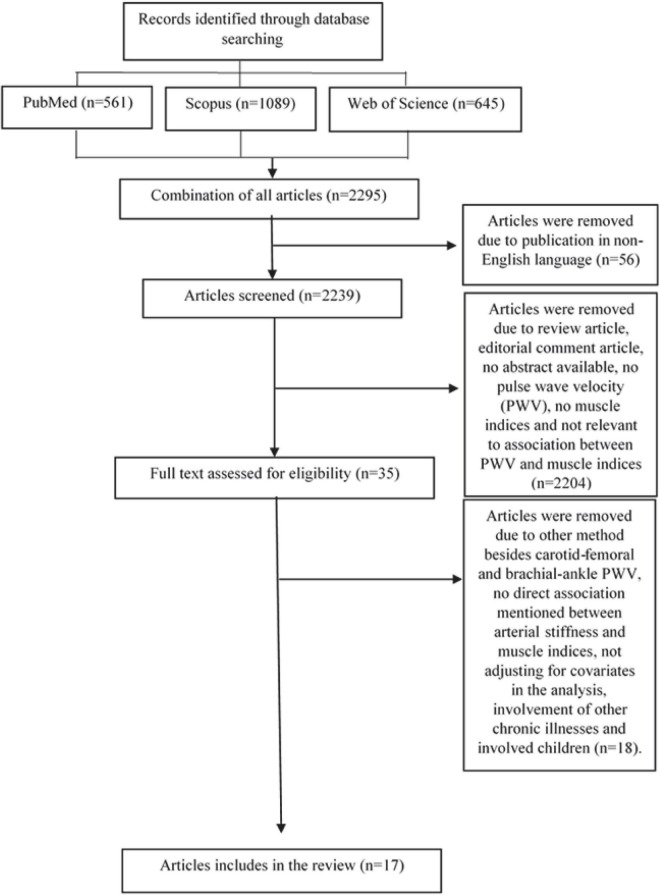
Flowchart of selection of the related articles.

From the three databases, a total of 2295 articles were obtained. These included 561 articles from PubMed, 1089 articles from Scopus, and 645 articles from Web of Science. The articles were published between the years 1971 and June 2021. A total of 56 articles written in languages other than English were omitted. After reading the titles or abstracts, 2204 articles were further omitted. The remaining 35 articles were obtained and reviewed thoroughly by fully reading the whole text. Out of these 35 articles, only 17 articles fulfilled the selection criteria, hence included in this review. The process of article selection is shown in Figure 1.

Table 1 summarized the two studies related to the association between muscle strength and AS in healthy subjects, while Table 2 summarized the seven studies related to the association between muscle strength and AS in subjects with established CVD and CVD risk factors. Table 3 summarized 11 studies related to the association between muscle mass and AS in subjects with CVD risk factors. There were no studies related to the association between muscle mass and AS in healthy subjects. The details of the parameters measured in each study were included in Tables 4, 5, respectively.

**TABLE 1:** Selected studies focusing on the association between arterial stiffness and muscle strength in healthy subjects.

References	Study design and subject characteristic	Mean age (years)	Male subjects (%)	Method		Correlation	
Fahs et al. (2010)	**Cross-sectional study** 79 young healthy men	23 ± 4	100	cfPWV by applanation tonometry (Millar Instruments, Houston, TX).	One-repetition maximum (1-RM) of the supine bench press.	After adjustment for age, BMI, SBP, and cardiorespiratory fitness, increased strength was associated with lower prevalence of high cPWV (odds ratio=0.14, 95% confidence interval= 0.02–0.92, *P* = 0.04).	Significant, negatively associated.
Gonzales (2013)	**Cross-sectional study** Participants aged 61–78 years old, healthy subjects and void of metabolic and cardiovascular diseases. Men (*n* = 9) Women (*n* = 12)	72 ± 5 65 ± 4	42.9	cfPWV using applanation tonometry with simultaneous ECG (SphygmoCor, Atcor Medical, Sydney, Australia).	Gait performance (speed and distance) measured by using a 400 m walk test completed in best time.	cfPWV was associated with 2-min walk distance (*r* = −0.51; *P*<0.05) and gait speed (*r* = −0.48; *P*<0.05) from partial correlation analysis controlling for age, body mass index, waist circumference and systolic blood pressure.	Significant, negatively associated

*BMI, body mass index; cfPWV, carotid femoral pulse wave velocity; ECG, electrocardiogram; SBP, systolic blood pressure.*

**TABLE 2:** Selected studies focusing on the association between arterial stiffness and muscle strength in subjects with cardiovascular diseases or cardiovascular diseases risk factors.

References	Study design and subject characteristic	Mean Age (years)	Male subjects (%)	Method		Correlation	
				PWV carotid femoral and brachial ankle	Muscle strength	PWV and strength	
Watson et al. (2011)	**Prospective study** 2,172 nondisabled men and women aged 70–79 years old from Pittsburgh, PA and Memphis, TN were followed up for 7 years Involved subjects with CVD, DM, HPT	73.6 ± 2.9	48.1	cfPWV using Doppler-recorded carotid and femoral pulse waveforms (model 810A, 9.0–10 MHz probes; Parks Medical Electronics, Aloha, OR).	Gait speed assessed by measuring time to walk a 20 m straight course.	In PAD patients (*n* = 261; 12.7%), PWV was associated with gait speed at baseline and throughout the study period after adjustment for demographics, risk factors, and chronic conditions [OR=-0.028, CI (–0.047, –0.010), *P* < 0.01].	Significant, negatively associated in PAD cohort. No significant association in full cohort.
van Dijk et al. (2015)	**Prospective/Interventional** 497 participants aged 65 years and older. Intervention involving the use of 500 μg vitamin B12 and 400 μg folic acid. Both the intervention and the placebo groups received 15 μg vitamin D. Inclusion criteria: age > 65 years and elevated homocysteine level (12–50 μmol/l). Involved subjects with CVD risk factors.	72.1 ± 5.4	57	cfPWV was measured using Sphygmocor device (Sphygmocor version 7.1, AtCor Medical, Sydney, Australia).	HGS using a strain-gauged dynamometer (Takei, TKK 5401, Takei Scientific Instruments Co., Ltd., Japan).	In the multiple linear regression, HGS was not associated with the amount of arterial stiffness after a follow-up period of 2 years. Adjusted for baseline measure of arterial stiffness parameter, age, gender, treatment allocation, study center, MAP, heart rate, smoking status, alcohol use, eGFR and BMI.	No significant association.
Rong et al. (2020)	**Cross-sectional study** 450 elderly people > 65 years old healthy. Sarcopenia (*n* = 89) Non-sarcopenia (*n* = 361) Involved subjects with CVD risk factors, cardiac disease.	72.48 ± 4.65 71.05 ± 4.15	56.18 59.83	baPWV; VP1000 (an automatic atherosclerosis tester produced by the Colin Company of Japan).	HGS using Jamar Hand dynamometer 5030 J1. (Lafayette Instrument Company, United States).	HGS was negatively associated with baPWV. Male (β = −6.132; *P* = 0.033) Female (β = −6.127; *P* = 0.043) Both (β = −8.007; *P* = 0.002). After adjusting for sex, age, BMI, VFA, hypertension, diabetes, cardiac, smoking, sports, MNA-SF, TG, LDL-C, HbA1C, Hb, ALB, and Cr	Significant, negatively associated.
Zhang et al. (2021)	**Cross-sectional study** 1046 healthy elderly subjects (>65 years old) Men, *n* = 448 Women, *n* = 598 Involved subjects with CVD risk factors.	71.7 ± 4.8 71.6 ± 4.5	42.8	baPWV: volume-plethysmographic apparatus (BP-203PRE II/III, Fukuda Colin Co., Ltd., Tokyo, Japan)	HGS: Smedley type digital grip dynamometer (T.K.K.5401, TAKEI Scientific Instruments Co., Ltd., Niigata, Japan).	baPWV was an independent predictor of HGS (β = −0.102, *P*< 0.001) after adjustment for age, sex, systolic BP, triglycerides, hemoglobin A1c, albumin, alcohol consumption, cigarette smoking, and sedentary behavior.	Significant, negative association.
(Lima-Junior et al., 2019)	**Cross-sectional study** 72 hypertensive patients on anti-hypertensive medications	58 ± 10	28	cfPWV (Sphygmocor, ATCOR Medical, Australia).	HGS using Digital dynamometer.	No significant association was observed between HGS and carotid femoral PWV in hypertensive patients (*P* > 0.05) after adjustment for related confounders.	No significant association.
Ohara et al. (2015)	**Cross-sectional study** 1593 middle-aged to older patients with no CVD events. 652 men 941 women Involved subjects with CVD risk factors.	65.7 ± 9.9 65.0 ± 8.9	40.93	baPWV using volume-plethysmograph (PWV/ABI; Omron Healthcare Co., Ltd.).	HGS: digital hand dynamometer (T.K.K. 5410; Takei Scientific Instruments Co., Ltd., Niigata, Japan).	HGS was significantly associated with baPWV for male (β = −0.11; *P* = 0.013) and female (β = −0.09; *P* = 0.011) with sarcopenia after adjustment for age, BMI, BP, HR, visceral fat, lipid profiles, CRP, smoking status, and medication used.	Significant, negatively associated.
Yamanashi et al. (2018)	**Cross-sectional study** Individuals aged = 40 years, consists of 1501 participants enrolled in the third follow-up of the Andhra Pradesh Children and Parents Study (APCAPS), India 703 Indian men 798 Indian women Involved subjects with CVD, stroke and CVD risk factors	53.5 ± 6.6 47.0 ± 5.3	47	baPWV using Vicoder system (Skidmore Medical Limited, Bristol, United Kingdom).	HGS; Lafayette Hand-held Dynamometer 78010 (Lafayette Instrument Company, Lafayette, Indiana, United States).	PWV was negatively associated with HGS for Indian men (β = −0.97; *P* = 0.001) and Indian women (β = −0.44; *P* = 0.020) in non-hypertensive group. In Indian men adjusted for age, height, body mass index (BMI), systolic blood pressure, albumin, history of ischemic heart disease, smoking status, daily energy intake and use of antihypertensive drugs. In Indian women adjusted for age, height, BMI, albumin, drinking status and use of antihypertensive drugs.	Significant, negatively associated in non- hypertensive subjects. Not significant in total and in hypertensive group.

*ALB, albumin, baPWV; brachial ankle pulse wave velocity; cfPWV, carotid femoral pulse wave velocity; HGS, hand grip strength; BMI, body mass index; SBP, systolic blood pressure; BW, body weight; Cr, creatinine; CRP, C-reactive protein; CVD, cardiovascular disease; eGFR, estimated glomerular filtration rate, Hb, hemoglobin; HbA1c, hemoglobin 1AC; HGS, handgrip strength, HR, heart rate; MAP, mean arterial pressure, MNA-SF, mini-nutritional assessment short-form; LDL, low-density lipoprotein; TG, triglyceride; T2DM, type-2 diabetes mellitus; VFA, visceral fat area; PAD, peripheral artery disease.*

**TABLE 3:** Selected studies focusing on the association between arterial stiffness and muscle mass in subjects with cardiovascular diseases or cardiovascular diseases risk factors.

References	Study design and subject characteristic	Mean age (years)	Male subjects (%)	Method		Correlation	
				PWV carotid femoral and brachial ankle	Muscle mass	PWV and mass	
Kim et al. (2011)	**Prospective observational cohort study** 510 adults enrolled in the Korean Sarcopenic Obesity Study Male (*n* = 191) Female (*n* = 319) Involved subjects with metabolic syndrome	52.2 ±14.4 51.±14.8	36.3	baPWV; volume-plethysmographic apparatus (model BP-203RPE II; Colin, Komaki, Japan)	MFR (g/cm^2^) =ASM(g)/VFA(cm^2^). ASM was evaluated with dual energy X-ray absorptiometry and VFA with computed tomography.	MFR was independently and negatively associated with baPWV (*P* = 0.002) after adjustment for age, BP and smoking (β=- 59.505)	Significant, negatively associated for MFR.
Zhang et al. (2021)	**Prospective study** 1046 elderly subjects (>65 years old) Men, *n* = 448 Women, *n* = 598 Involved subjects with CVD risk factors	71.7 ± 4.8 71.6 ± 4.5	42.8	baPWV: volume-plethysmographic apparatus (BP-203PRE II/III, Fukuda Colin Co., Ltd., Tokyo, Japan)	ASMI: BIA (Physion MD, Physion Co., Ltd., Kyoto, Japan).	baPWV was correlated with ASMI (β = −0.27, *P*< 0.001) after adjustment for age, sex, systolic BP, triglycerides, hemoglobin A1c, albumin, alcohol consumption, cigarette smoking, and sedentary behavior.	Significant negatively associated.
Ochi et al. (2010)	**Cross-sectional study** 496 middle-aged to elderly participants Involved subjects with CVD risk factors	Not stated	Not stated	baPWV: volume-plethysmographic apparatus (form PWV/ABI; Omron Healthcare Co., Ltd., Kyoto, Japan).	Thigh muscle CSA: CT image (lightSpeed VCT, GE Healthcare, Tokyo, Japan). Sarcopenic index: CSA corrected by body weight (BW).	BaPWV was associated with CSA/BW (β = −0.24; *P*< 0.01) in men after correction with age, height, BP, lipids, glucose level, antihypertensive medication, hs-CRP, smoking, alcohol consumption and physical activity.	Significant, negatively associated in men.
Ohara et al. (2015)	**Cross-sectional study** 1593 middle-aged to older patients with no history of symptomatic cardiovascular events. 652 men 941 women Involved subjects with CVD risk factors	65.7 ± 9.9 65.0 ± 8.9	40.93	baPWV was measured using a volume plethysmograph (PWV/ABI; Omron Healthcare Co., Ltd.).	1. Thigh muscle CSA: CT (LightSpeed VCT; GE Health- care, Tokyo, Japan) 2. Total skeletal muscle ratio: bioelectrical impedance method using body composition analyzer (body scan HBF-701; Omron Healthcare Co., Ltd., Kyoto, Japan).	Skeletal muscle mass was significantly associated with baPWV for male (β = −0.18; *P* = 0.0002) and female (β = −0.11; *P* = 0.0017) after adjustment for age, BMI, BP, HR, visceral fat, lipid profiles, glucose, hs-CRP, smoking status and medication used.	Significant, negatively associated.
Zhang et al. (2019)	**Cross-sectional study** 1002 Chinese elderly subjects aged above 65 years old Involved subjects with CVD risk factors	72.3 ± 5.2	41.9	baPWV using Vascular Profiler-1000 device (Omron, Kyoto, Japan).	Body composition (BIA; InBody 770; Biospace Co., Ltd., Seoul, Korea).	BaPWV was associated with ASMI (OR, 1.11; 95% CI, 1.04–1.20, *P*< 0.01) Adjustment applied for sex, age, body mass index, smoking, drinking, mean blood pressure, heart rate, the serum total-to-HDL cholesterol ratio, glycosylated hemoglobin and carotid intima-media thickness, hypertension, diabetes mellitus, and stroke.	Significant, negatively associated.
Rong et al. (2020)	**Cross-sectional study** 450 elderly people aged above 65 years old who received general medical examinations in Tianjin First Center Hospital, could walk by themselves without using a walking aid during gait speed measurement and did not have illness. Sarcopenia (*n* = 89) Non-sarcopenia (*n* = 361) Involved subjects with CVD risk factors	72.48 ± 4.65 71.05 ± 4.15	56.18 59.83	baPWV using VP-1000 plus.	ASMI =(ASM)/height(m^2^). ASM (BIA, InBodyS10, InBody Japan Inc., Tokyo, Japan).	After adjustment for sex, age, BMI, VFA, hypertension, diabetes, cardiac disease, smoking, sports, MNA-SF, TG, LDL-C, HbA_1C_, Hb, ALB and Cr, ASMI was negatively associated with baPWV in men (β = −32.752; *P*< 0.0001) women (β = −30.653; *P*< 0.0001) and both (β = −39.783; *P*< 0.0001)	Significant, negatively associated.
Yang et al. (2020)	**Cross-sectional study** 20,477 Chinese aged 45–80 years old Men (*n* = 6,390) Women (*n* = 14,087) Involved subjects with CVD risk factors	62.87 ± 8.07 60.30 ± 8.00	31.2	baPWV using plethysmography apparatus (BP-203RPE III; Omron, Japan).	Skeletal muscle mass measured using Dual bioelectrical impedance analyzer (IOI 353; Jawon, Korea).	After adjustment for age, body fat percentage, and BP, ASMI was negatively associated with baPWV [β (SE) for men: –0.208 (0.016), *P*< 0.0001; for women: –0.245 (0.012), *P*<0.0001].	Significant, negatively associated.
Tanaka et al. (2016)	**Cross-sectional study** 97 Japanese postmenopausal women with T2DM.	65.2 ± 8.9	0	baPWV was measured by using VaSera VS-1000 (Fukuda Denshi Tokyo, Japan).	ASM was measured using whole body DXA (QDR-4500, Hologic Co., Bedford, MA). RSMI was calculated using this formula: ASM/height^2^.	RSMI was significantly and negatively associated with baPWV (β = −0.40; *P* = 0.027) after adjustment for age, duration of T2DM, systolic BP, BMI, HbA1c, serum creatinine, LDL-C, and uric acid as well as the usage of anti-hypertensive.	Significant, negatively associated.
Fischer et al. (2021)	**Cross-sectional study** 93 postmenopausal women without cardiovascular, pulmonary, musculoskeletal disease and not smoking Involved subjects with CVD risk factors	59 ± 5	0	baPWV was measured by automatic device (VP 2000; Omron, Kyoto, Japan)	Lean mass of the total body, arms (ArmLM), legs (LegLM), and trunk were measured using a DXA (GE Lunar DPX-IQ, Madison, WI). ASMI was calculated as the combined ArmLM and LegLM (kg) divided by the height in meters squared (ArmLM+LegLM)/Ht^2^).	After adjusting for age, height, brachial SBP, MVC and HR, baPWV was correlated with ASMI (β = −0.23, *P* = 0.043) and ArmLM (β = −0.23, *P* = 0.045). No association with LegLM (β = −0.19, *P* = 0.074).	Significant, negatively associated.
Inomoto et al. (2021)	**Cross-sectional study** 1403 male workers: Aged 25–34 (*n* = 217) Aged 35–44 (*n* = 359) Aged 45–54 (*n* = 457) Aged 55–64 (*n* = 370) Involved subjects with CVD risk factors	28.8 ± 2.9 40.1 ± 2.8 49.4 ± 3.0 59.1 ± 2.8	100	baPWV: BP pulse wave tester (BP-203RPEIII, Fukuda Colin Co., Ltd., Tokyo, Japan).	Skeletal muscle index: BIA (InBody 720, InBody Co., Ltd., Seoul, Korea).	Skeletal muscle index was an independent variable for baPWV in workers aged 35–44 and 45–54 years old (Standardized coefficient = −0.164 and −0.143 respectively, *P*< 0.01 for both) after adjustment for smoking, BP, HR and physical activity in 45–54 years old and smoking, BP and HR in 35–44 years old.	Significant, negatively associated.
Yoon et al. (2020)	**Cross-sectional study** 1,710 adults >20 years of age who visited a Health Promotion Centre in South Korea for a health check-up between January 2017 and July 2019. Male Female Involved subjects with CVD risk factor	51.8 ± 12.3 52.5 ± 13.5	72	baPWV was measured using a volume-plethysmography apparatus (BP-203RPE III, Omron Healthcare Co.; Kyoto, Japan).	Skeletal muscle mass index (SMI) was estimated with a multi-frequency BIA (InBody 720, Biospace Co., Seoul, Korea).	SMI was negatively correlated with baPWV in the male population (β = −0.188, *P*< 0.001) and female (β = −0.136, *P* = 0.011) after adjusting for age, comorbidities, BMI, lipid levels, smoking, glucose level, alcohol consumption, exercise and menopause status.	Significant negatively associated.

*ALB, serum albumin; ASM, appendicular skeletal muscle mass; ASMI, Appendicular skeletal muscle index; BIA, bioelectrical impedance analysis; BMI, body mass index; BP, blood pressure; BW, body weight; Cr, creatinine; CRP, C-reactive protein; CSA, cross sectional area; CVD, cardiovascular disease; DXA, dual-energy X-ray absorptiometry; Hb, hemoglobin; Hb1AC, hemoglobin 1AC; HDL, high-density lipoprotein; HR, heart rate; MNA-SF, mini-nutritional assessment short-form; MFR, muscle-to-fat ratio; MVC, maximal voluntary contractions; LDL, low-density lipoprotein; RSMI, Relative skeletal muscle mass index; SMI, skeletal muscle index; TG, triglyceride; T2DM, type-2 diabetes mellitus; VFA, visceral fat area.*

**TABLE 4:** Values of related parameters in each study (muscle strength).

References	Subjects	Muscle strength	PWV value		SBP/DBP (mmHg)	HR (bpm)	BMI (kg/m^2^)
Watson et al. (2011)	Non-disabled older men and women	Gait speed (m/s) 1.34 ± 0.25	3.12–29.98 m/s		135.5 ± 19.3/71.6 ± 10.9	64.5 ± 10.6	27.3 ± 4.7
van Dijk et al. (2015)	497 older individuals	33.1 ± 10.2 kg (baseline)	Baseline 14.1 ± 4.3 m/s	Follow up after 2 years 14.2 ± 4.4 m/s	137.1 ± 17.8/77.2 ± 9.4	Not stated	27.0 ± 3.7
Lima-Junior et al. (2019)	Hypertensive subjects	31 ± 10 kg	8.8 ± 1.9 m/s		132 ± 16/74 ± 10	68 ± 11	30.6 ± 5.5
Rong et al. (2020)	Sarcopenia	23.99 ± 5.60 kg	17.92 ± 12.8 m/s		Not stated	Not stated	24.01 ± 1.92
	Non-sarcopenia	25.48 ± 5.72 kg	16.48 ± 12.1 m/s				25.20 ± 2.31
Ohara et al. (2015)	Men	39.3 ±6.8 kg	16.39 ± 3.31 m/s		137.1 ± 19.1/78.7 ± 11.3	64.9 ± 10.3	24.0 ± 2.9
	Women	23.6 ±4.3 kg	15.47 ± 3.30 m/s		133.0 ± 19.7/75.4 ± 11.0	67.1 ± 9.7	22.8 ± 3.1
Fahs et al. (2010)	Young, healthy men	95.0 ± 30.4 kg	5.9 ± 0.7 m/s		126 ± 9/74 ± 7	58 ± 9	26.5 ± 4.7
Yamanashi et al. (2018)	Indian men	25.4 ± 7.1 kg	8.04 ± 1.32 m/s		130.0 ± 21.4/85.9 ± 15.0		20.5 ± 3.7
	Indian women	17.5 ± 4.9 kg	7.62 ± 1.23 m/s		123.3 ± 16.2/81.4 ± 11.5	Not stated	17.5 ± 4.9
Gonzales (2013)	Subjects void of metabolic and cardiovascular diseases	Gait speed (m/s)					
	Men	1.5 ± 0.3	8.9 ± 2.7 m/s		117 ± 9/73 ± 7	Not stated	24 ± 2
	Women	1.5 ± 0.2	9.2 ± 2.4 m/s		119 ± 15/74 ± 11		23 ± 3
Zhang et al. (2021)	Elderly subjects						
	Men	33.5 ± 6.2 kg	17.57 ± 3.50 m/s		131 ± 17/74 ± 10	Not stated	23.1 ± 3.1
	Women	21.1 ± 4.0 kg	16.97 ± 3.20 m/s		130 ± 18/75 ± 10		22.6 ± 3.3

*BMI, body mass index; DBP, diastolic blood pressure; HR, heart rate; PWV, pulse wave velocity; SBP, systolic blood pressure.*

**TABLE 5:** Values of related parameters in each study (muscle mass).

References	Subjects	Muscle mass		PWV value	SBP/DBP (mmHg)	HR (bpm)	BMI (kg/m^2^)
Ohara et al. (2015)	Men	18.8 ± 3.2 kg		16.39 ± 3.31 m/s	137.1 ± 19.1/ 78.7 ± 11.3	64.9 ± 10.3	24.0 ± 2.9
	Women	12.2 ± 2.0 kg		15.47 ± 3.30 m/s	133.0 ± 19.7/ 75.4 ± 11.0	67.1 ± 9.7	22.8 ± 3.1
Zhang et al. (2019)	Distribution of BaPWV: <15.61	6.89 ± 0.98 kg/m^2^		Not stated	126.5 ± 15.2 / 72.5 ± 9.4	70.8 ± 10.3	24.6 ± 3.3
	15.61–17.33	6.77 ± 0.96 kg/m^2^			132.7 ± 14.4 / 72.9 ± 8.8	71.7 ± 10.2	24.9 ± 3.2
	17.33–19.74	6.65 ± 0.93 kg/m^2^			138.9 ± 14.4 / 74.6 ± 9.9	72.8 ± 10.9	24.7 ± 3.3
	>19.74	6.44 ± 0.94 kg/m^2^			144.7 ± 18.1 / 76.0 ± 10.8	77.8 ± 12.0	24.7 ± 3.6
Rong et al. (2020)	Sarcopenia Non-sarcopenia	6.59 ± 0.73 kg/m^2^ 7.64 ± 0.76 kg/m^2^		17.92 ± 12.8 m/s 16.48 ± 12.1 m/s	Not stated	Not stated	24.01 ± 1.92 25.20 ± 2.31
Yang et al., 2020)	Men Women	49.36 ± 6.18 kg 38.13 ± 4.32 kg		16.50 m/s 15.67 m/s	137.41 ± 19.0/ 81.32 ± 10.95 135.87 ± 20.63/ 78.34 ± 11.14	Not stated	24.51 ± 3.23 24.39 ± 3.40
Tanaka et al. (2016)	Postmenopausal women with T2DM	6.38 ± 1.08 kg/m^2^		15 ± 2.8 m/s	129.0 ± 19.5/ 75.0 ± 11.1	Not stated	24.3 ± 5.2
Ochi et al. (2010)	Apparently healthy subjects	–		–	–	–	–
Fischer et al. (2021)	Postmenopausal women	ASMI = 7.45 ± 1.4 (kg/m2) Leg lean mass = 15.1 ± 3.2 (kg) Arm lean mass = 4.9 ± 1.2 (kg)		15.1 ± 2.0 m/s	138 ± 14/ 80 ± 8	–	30.8 ± 6.6
Inomoto et al. (2021)	Male workers Aged 25–34 Aged 35–44 Aged 45–54 Aged 55–64	7.9 ± 0.7 kg/m^2^ 8.1 ± 0.7 kg/m^2^ 8.1 ± 0.6 kg/m^2^ 7.9 ± 0.6 kg/m^2^		11.80 ± 12.3 m/s 12.83 ± 16.8 m/s 13.48 ± 19.7 m/s 14.95 ± 24.3 m/s	124.5 ± 11.4/ 72.1 ± 8.2 130.2 ± 14.2/ 78.8 ± 10.4 133.8 ± 15.4/ 83.3 ± 11.4 136.3 ± 17.2/ 84.0 ± 11.2	67.7 ± 11.4 71.5 ± 11.8 70.7 ± 12.3 69.9 ± 11.4	23.7 ± 4.1 24.8 ± 3.7 24.6 ± 3.1 24.0 ± 2.9
Zhang et al. (2021)	Elderly subjects Men Women	4.8 ± 0.6 kg/m^2^ 4.1 ± 0.5 kg/m^2^		17.57 ± 3.50 m/s 16.97 ± 3.20 m/s	131 ± 17/ 74 ± 10 130 ± 18/ 75 ± 10	74 ± 10 75 ± 10	23.1 ± 3.1 22.6 ± 3.3
Yoon et al. (2020)	Adults >20 years of age Male Female	10.4 ± 1.0 kg/m^2^ 8.3 ± 0.8 kg/m^2^		14.33 ± 2.93 m/s 13.86 ± 2.98 m/s	127.3 ± 13.9/ 75.7 ± 10.1 123.5 ± 15.0/ 72.9 ± 8.9	Not stated	24.9 ± 3.1 23.7 ± 3.2
Kim et al. (2011)	Adults >20 years of age Male Female	ASMI 9.2 ± 1.0 kg/m^2^ 7.5 ± 0.9 kg/m^2^	MFR 199.8 [151.5,247.9] g/cm^2^ 195.7 [134.0,298.5] g/cm^2^	14.09 ± 3.01 m/s 13.14 ± 2.62 m/s	125.9 ± 12.4/ 82.9 ± 9.8 119.9 ± 13.9/ 77.3 ± 9.9	Not stated	25.2 ± 3.1 23.9 ± 3.7

*ASMI, Appendicular skeletal muscle index; BMI, body mass index; DBP, diastolic blood pressure; EFNW, excessive fat normal weight; EFO, excessive fat obese; HR, heart rate; MFR, muscle-to-fat ratio; NFO, normal fat obese; NFNW, normal fat normal weight; PWV, pulse wave velocity; SBP, systolic blood pressure. Data is expressed in median [inter-quartile range].*

For the association between muscle strength and AS in healthy subjects (Table 1), all studies found that muscle strength was inversely correlated with arterial stiffness as measured by cfPWV (Fahs et al., 2010; Gonzales, 2013). A cross-sectional study showed that increased muscle strength was associated with lower prevalence of high cfPWV in young healthy men (odds ratio = 0.14, 95% confidence interval = 0.02–0.92, *P* = 0.04) (Fahs et al., 2010). Another study by Gonzales (2013) demonstrated that cfPWV was associated with the gait distance in healthy, older subjects (*r* = −0.51; *P* < 0.05) (Gonzales, 2013).

In subjects with CVD or CVD risk factors (Table 2), five out of seven studies found significant associations (Watson et al., 2011; Ohara et al., 2015; Yamanashi et al., 2018; Rong et al., 2020; Zhang et al., 2021). For example, the study by Rong et al. (2020) found that muscle strength as measured by handgrip dynamometer was negatively associated with baPWV in elderly subjects (Rong et al., 2020). Whereas the study by Ohara et al. (2015) found that muscle strength as measured by handgrip dynamometer was negatively associated with baPWV in middle-aged and older subjects (Ohara et al., 2015). A prospective study found a significant association between aortic PWV and gait speed in peripheral arterial disease (PAD) patients (OR = −0.028, CI (−0.047, −0.010), *P* < 0.01), but the association was not significant in total cohort population (Watson et al., 2011). Similarly, the study by Yamanashi et al. (2018) found an inverse association between handgrip strength and PWV in non-hypertensive adults [men (β = −0.97; *P* = 0.001), women (β = −0.44; *P* = 0.020)], but not in the hypertensive group and the total cohort (Yamanashi et al., 2018). Another two studies did not observe any significant association between AS and muscle strength (van Dijk et al., 2015; Lima-Junior et al., 2019).

As for the association between muscle mass and PWV, all 11 relevant studies involved subjects with CVD or CVD risk factors. Most of the studies were cross-sectional studies. All the eleven studies found a negative association between muscle mass and PWV (Ochi et al., 2010; Kim et al., 2011; Ohara et al., 2015; Tanaka et al., 2016; Zhang et al., 2019, 2021; Rong et al., 2020; Yang et al., 2020; Yoon et al., 2020; Fischer et al., 2021; Inomoto et al., 2021). Five studies that measured the appendicular skeletal muscle index (ASMI) as the muscle mass marker showed a negative association between ASMI and baPWV (Zhang et al., 2019, 2021; Rong et al., 2020; Yang et al., 2020; Fischer et al., 2021). For example, the study by Rong et al. (2020) found that ASMI was negatively associated with baPWV in elderly Chinese after adjustment of potential confounders [(β = −32.752; *P* < 0.0001 (men); β = −39.783; *P* < 0.0001) (women)] (Rong et al., 2020).

Yang et al. (2020) found that low muscle mass was associated with increased risk of AS in Chinese nationals aged 45 years old and older (men, *P* ≤ 0.0001, β = −0.208, women, *P* ≤ 0.0001, *β* = −0.245) (Yang et al., 2020). In Japan, subjects older than 65 years old showed a negative association between baPWV and ASMI (β = −0.27; *P* < 0.001) (Zhang et al., 2021). A study on post-menopausal women also revealed similar finding whereby ASMI and arm leg mass (armLM) were negatively associated with baPWV with β = −0.23 (*P* = 0.043) and β = −0.23 (*P* = 0.045), respectively (Fischer et al., 2021). A cross-sectional study by Kim et al. (2011) observed that appendicular skeletal muscle mass (ASM) was not associated with baPWV. However, there was a significant association between ASM and visceral fat area ratio (MFR) (β = −59.505, *P* = 0.002) (Kim et al., 2011).

Relative skeletal muscle mass (calculated using the formula ASM/height^2^) had a negative association with baPWV in Japanese postmenopausal women with type 2 diabetes mellitus (T2DM) (Tanaka et al., 2016). Ohara et al. (2015) also found that skeletal muscle mass was inversely associated with baPWV in male (β = −0.18; *P* = 0.0002) and female subjects (β = −0.11; *P* = 0.0017) (Ohara et al., 2015). Skeletal muscle index is another measurement of muscle mass used in two studies. South Korean men and women showed a negative association between their skeletal muscle index and baPWV [(β = −0.188; *P* < 0.001 (men); β = −0.136; *P* = 0.011) (women)], whereas working men with low muscle mass in Japan (aged 35–44 and 45–54 years old) had a higher risk of AS (Yoon et al., 2020; Inomoto et al., 2021).

## Discussion

Arterial compliance represents the capacity of the artery to expand and recoil following heart contraction and relaxation, which permits blood flow from pulsatile and intermittent form to a steadier, laminar flow (Sherwood, 2008). Increased AS give more resistant to the blood flow and higher workload for the left ventricle. This led to increased blood pressure and enhanced atherosclerosis development (Oren et al., 2003; Mitchell et al., 2005).

The main factors contributing to AS are aging and atherosclerosis (Greenland et al., 2010). The artery becomes stiff when the collagen in the arterial wall increased and the elastin tissue decreased (Zieman et al., 2005). Besides structural changes, increase in local vasoconstrictor such as endothelin-1 (ET-1) or reduction in vasodilator such as nitric oxide (NO) may contribute to AS (Bellien et al., 2010; Guo et al., 2018). These modulators are released from vascular endothelial cells and has a significant role in the control of vascular activity (Santos-Parker et al., 2017; Trindade et al., 2017). Poor NO production is a cardinal feature of endothelial dysfunction (ED) (Sun et al., 2020). Several CAD risk factors such as hypertension, dyslipidemia and diabetes mellitus were known to increase AS through underlying ED (Rider et al., 2012; Aminuddin et al., 2020; Ji et al., 2020). Thus, having CAD risk factors would accelerate AS on top of the aging process.

In our review, it was found that in most of the studies, muscle indices had negative association with AS as measured by PWV. This association is evident in both healthy subjects and subjects with CVD or CVD risk factors. However, most of the studies are cross-sectional studies, hence the studies were unable to determine the cause-effect association between muscular functions and AS. There were only two prospective studies that determined the direct relationship between AS and muscle indices (Watson et al., 2011; van Dijk et al., 2015). Watson et al. (2011) showed that in PAD patients, higher PWV was independently associated with slower gait speed, thus suggesting that increased PWV leads to reduction in muscle strength. In contrast, van Dijk et al. (2015) showed that hand-grip strength was not associated with AS after a follow-up duration of 2 years. The author proposed that the lack of an association might be due to the difference between short- and long-term alteration of the arteries. It has been shown that structural adaptations of the artery such as changes in elastin and collagen need a longer time to develop (van Dijk et al., 2015). Another cross-sectional study by Lima-Junior et al. (2019) also showed no significant association between PWV and muscle indices. Small sample size might account for such discrepancy (Lima-Junior et al., 2019).

Regular exercise is an important modality in improving cardiovascular health and endothelial function (Francescomarino et al., 2009; Aminuddin et al., 2011), thus improving AS (Kollet et al., 2021). Exercise also increases muscle strength (Chen et al., 2017; Sanian et al., 2019; Otsuki et al., 2020). Increased muscle strength and reduced AS were inversely related with CVD risk factors such as increased levels of low-density lipoprotein, body fat and inflammation, decreased lean tissue mass and insulin resistance (Fahs et al., 2010; Nishitani et al., 2011; Rider et al., 2012; Farias et al., 2013; Aminuddin et al., 2020; Ji et al., 2020).

In terms of the association between AS and muscle mass, previous studies showed that there were negative association between low muscle mass and AS. There are several mechanisms that can explain such relationship. Firstly, an increase in AS may reduce basal limb blood flow, leading to decreased delivery of nutrients and oxygen to the muscle tissues and lower muscle mass (Suzuki et al., 2001; Abbatecola et al., 2012; Rong et al., 2020). AS is also associated with increased reflected wave, systolic blood pressure and pulse pressure along the arterial system that cause small vessel injury (Safar et al., 2003). Secondly, the muscle mass itself may exert an effect on AS. When there is muscle death due to muscle disuse or aging, maladaptive muscle remodeling may occur if there is impaired removal of the apoptotic cells (Siu et al., 2005; Sciorati et al., 2016). This includes fatty infiltration within the muscles that leads to the release of inflammatory cytokines (Sciorati et al., 2016). Inflammatory cytokines such as tumor necrosis factor-α, interleukin (IL)-1β and IL-6 activate several inflammatory signaling pathways that promote insulin resistance (Chen et al., 2015). Inflammation also causes proteolysis which leads to reduced muscle mass (Goodman, 1991). In addition, reduced muscle mass is also associated with insulin resistance, since skeletal muscle is the major site of glucose utilization (Yamanashi et al., 2018). Insulin resistance has been linked to AS (Ikonomidis et al., 2015), which could be explained by the underlying reduction in NO bioavailability (Sonne et al., 2009), increased endothelin levels and increased proliferation of vascular smooth muscle cells (Trovati and Anfossi, 2002).

**FIGURE 2:**
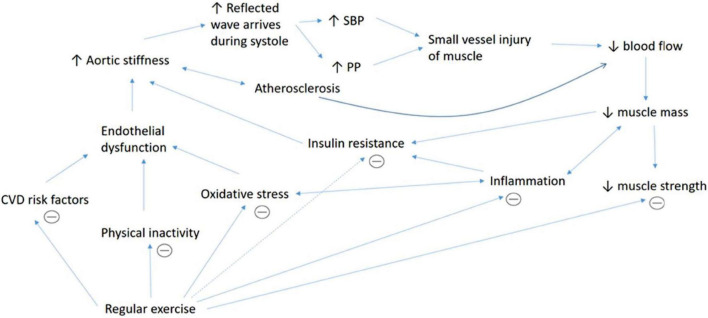
Summary of the underlying mechanisms that explain the association between AS and muscle indices. The association between aortic stiffness and muscular indices is complex, involves multiple intermediators and may act in a vicious cycle. Increased aortic stiffness leads to increased wave reflection and augmentation of systolic blood pressure (SBP) and pulse pressure (PP), which causes injury to small vessels in the organ such as muscle. Aortic stiffness which is also associated with atherosclerosis causes reduced blood supply to the muscle that impairs nutrient supplementation. This contributes to muscle death and reduced muscle mass. Reduced muscle mass also contributes to lower muscle strength. Besides, lower muscle mass causes less glucose intake into the muscle cells, which leads to insulin resistance. Poor muscle removal after cell death leads to fat infiltration and inflammation. Increased inflammation itself may cause proteolysis, reduced muscle mass, insulin resistance and increased oxidative stress. Oxidative stress causes proteolysis by causing myocardial DNA damage and stimulating the release of various inflammatory mediators. Inflammation, oxidative stress, various cardiovascular diseases (CVD) risk factors and physical inactivity are linked to endothelial dysfunction, which leads to increased aortic stiffness. Regular physical activity increases muscle mass and strength and improves endothelial function, oxidative stress, CVD risk factors, insulin resistance and inflammation which subsequently reduces aortic stiffness.

Another dynamic that links muscle mass and AS is through increased oxidative stress. In this case, muscle mass may not directly affect AS and vice versa, but rather a distinct complication of a common factor which is oxidative stress. Oxidative stress happens when there is an imbalance between the antioxidant and free radicals in the body that leads to damage to the cellular protein, lipid and nucleic acids (Wu et al., 2013). Oxidative stress is related to sarcopenia (Bellanti et al., 2018) through modulations of transcription factors and inflammatory mediators such as nuclear factor-κB, Forkhead box (FOXO) and mitogen-activated protein kinase that lead to muscle apoptosis and reduced protein synthesis. Besides, oxidative stress causes myocardial DNA damage and dysfunction which later leads to muscle apoptosis and sarcopenia (Meng and Yu, 2010). Additionally, oxidative stress is related to the formation of AS through reduction in NO (Zhao et al., 2011; Förstermann et al., 2017; Guzik and Touyz, 2017). The proposed, complex mechanisms that explain the association between AS and muscle indices are summarized in Figure 2.

The strength of this study is that we focused on the established markers of PWV which are cfPWV and baPWV. However, there are certain limitations of this study that include (1) the cross-sectional design of the selected studies, in which cause-effect associations between muscle indices and arterial stiffness could not be determined, and (2) the absence of studies related to AS and muscle flexibility, which is one of important markers for muscular functions. On the other hand, we also excluded several studies that reported significant associations between AS and muscle indices that were derived from simple correlations without adjustment for confounders (Yamamoto et al., 2009; Komatsu et al., 2017; Logan et al., 2018). Thus, future studies should be conducted with a detail analysis adjusted for the covariates to address such associations.

## Conclusion

There is an inverse association between muscle indices and AS in healthy subjects and subjects with established CVD and CVD risk factors. However, most of the studies reviewed are cross-sectional studies, hence no causal relationship or explanatory capacity between muscle indices and AS could be established. Therefore, more prospective studies should be conducted in the future to determine the interaction between muscle indices and AS.

## Author Contributions

BC and NJ performed the screening of articles. AA, MN, NM, and LZ drafted the manuscript. AA and AU finalized the manuscript. NC and NS contributed to the revision and editing of the manuscript. All authors read and approved the final manuscript.

## Conflict of Interest

The authors declare that the research was conducted in the absence of any commercial or financial relationships that could be construed as a potential conflict of interest.

## Publisher’s Note

All claims expressed in this article are solely those of the authors and do not necessarily represent those of their affiliated organizations, or those of the publisher, the editors and the reviewers. Any product that may be evaluated in this article, or claim that may be made by its manufacturer, is not guaranteed or endorsed by the publisher.
